# Healthcare Professionals’ Perspectives on Barriers and Facilitators to Medication Adherence Post Myocardial Infarction: A Qualitative Study Using the Theoretical Domains Framework

**DOI:** 10.3390/pharmacy14010023

**Published:** 2026-02-02

**Authors:** Fatma El-Komy, Michelle O’Driscoll, Stephen Byrne, Margaret Bermingham, Laura J. Sahm

**Affiliations:** 1 Pharmaceutical Care Research Group, School of Pharmacy, University College Cork, T12 YN60 Cork, Irelandl.sahm@ucc.ie (L.J.S.); 2 Pharmacy Department, Mercy University Hospital, Grenville Place, T12 WE28 Cork, Ireland

**Keywords:** medication adherence, myocardial infarction, healthcare professionals, health knowledge, patient education, barriers, facilitators

## Abstract

Medication adherence following myocardial infarction (MI) is essential for effective secondary prevention, yet adherence rates remain suboptimal. Healthcare professionals (HCPs) are central to promoting adherence through clinical decision-making, patient education, and ongoing behavioural support. Understanding how HCPs perceive and experience the factors’ influencing adherence is key to developing effective, context-specific interventions. This study explored HCPs’ perspectives on medication adherence post-MI and identified behavioural determinants influencing medication management across the care pathway. A qualitative descriptive study was conducted using semi-structured interviews with HCPs in the southwest of Ireland. Participants included hospital pharmacists, community pharmacists, general practitioners (GPs), cardiologists, and nurses, recruited through purposive, convenience, and snowball sampling. Interviews were recorded, transcribed verbatim, and analysed using directed content analysis guided by the Theoretical Domains Framework (TDF). Twelve HCPs (eight female) were interviewed between December 2024 and May 2025, including four pharmacists, two GPs, three cardiologists and three nurses. Interviews lasted 30–50 min (mean 41 min). Analysis identified 15 facilitators, 13 barriers, and 7 dual-role determinants across 10 TDF domains. Novel contributions include demonstrating how HCPs’ real-world experiences contextualise adherence issues in the distinct post-MI setting characterised by abrupt care transitions, polypharmacy, and emotional vulnerability and identifying where HCPs feel most constrained and where their expertise could directly inform targeted intervention design. HCPs’ insights reveal complex, context-specific behavioural determinants influencing post-MI medication adherence and highlight the need for multidisciplinary, tailored, and system-level solutions. Enhancing collaboration, supporting patient-centred communication, and addressing resource barriers could empower HCPs to deliver more effective, personalised adherence support and inform the development of targeted intervention strategies.

## 1. Introduction

The World Health Organisation (WHO) estimates that only half of people with chronic conditions adhere to prescribed treatments, with even lower rates reported in cardiovascular disease [1]. After myocardial infarction (MI), non-adherence to cardioprotective medication remains a major barrier to secondary prevention. Despite the proven efficacy of these medications, which include, e.g., statins and antiplatelets, fewer than half of patients remain adherent two years post-discharge, with discontinuation linked to increased risk of recurrent events, hospitalisation, and mortality [2,3].

Previous research has identified multiple barriers and facilitators to medication adherence post-MI. A recent qualitative systematic review shows that lack of knowledge about medications, beliefs about their consequences, and environmental context and resources, such as communication and follow-up challenges, can serve as barriers to adherence, while social support and positive beliefs about treatment can act as facilitators for sustained medication use [4]. Health system factors such as reduced co-payments, full prescription coverage, and patient counselling have also been reported to improve long-term adherence in secondary prevention settings, highlighting the importance of both individual and system-level influences [5].

Healthcare professionals (HCPs) are central to addressing medication management challenges [6]. In Ireland, post-MI care typically involves hospital admission under the care of a cardiology team, followed by cardiac rehabilitation and general practitioner (GP)-led community management with input from practice nurses and pharmacists. Cardiac rehabilitation, considered the standard of care, is delivered by multidisciplinary teams including cardiologists, nurses, physiotherapists, dietitians, psychologists, and pharmacists. This model combines structured exercise, behavioural counselling, and psychosocial support to promote recovery, risk factor modification, medication adherence, and well-being [7,8,9]. The Irish healthcare system has introduced a national Chronic Disease Management Programme in general practice, which supports structured, proactive care for people with conditions including cardiovascular disease. The Chronic Disease Management programme is available to people with public healthcare coverage and includes regular reviews, care planning, and integrated team-based approaches that aim to reduce hospitalisations and improve disease control (Health Service Executive [HSE], 2020) [10].

Effective adherence depends on the therapeutic alliance between patients and providers. Trust, communication, and continuity of care consistently result in better outcomes, while personalised approaches tailored to the patient’s health literacy, culture, and preferences outperform standardised strategies [11,12,13]. Shared decision-making (SDM) further empowers patients to engage with treatment and reduces the likelihood of recurrent events [14]. To improve long-term medication adherence post-MI, the relationship between the patient and HCPs is crucial [15]. Optimising the therapeutic partnership between patients and HCPs is critical in achieving effective secondary prevention. Given the close relationship between HCPs and their patients, a greater understanding of HCPs’ perspectives on the factors they believe influence medication adherence is essential. This will enable the development of targeted, evidence-based interventions and strategies that are likely to be both effective and adopted by HCPs [16,17,18]. While many studies have explored patient-reported barriers to medication adherence, less attention has been given to the perspectives of HCPs [19,20,21]. Considering the views of both patients and HCPs is crucial, as adherence is shaped not only by individual behaviours but also by the systems, practices, and relationships through which care is delivered. HCPs play a central role in initiating, monitoring, and sustaining long-term pharmacotherapy, and their insights are essential for identifying feasible, practice-based strategies that complement patient perspectives.

Research exploring post–MI medication adherence has tended to focus on individual barriers and facilitators, while giving less attention to the perspectives of the full range of healthcare professionals involved in patient care. In particular, the experiences of those working across the transition from hospital to community settings remain underrepresented in the literature. Although previous studies have generated valuable descriptive insights, there has been limited use of behaviour change theory or implementation frameworks to help translate these findings into practical, context-specific changes in care delivery. There is a clear need for research that moves beyond description to examine how and where meaningful change can be achieved within real-world healthcare systems. Greater attention is also required to understand how professional roles, system pressures, and interprofessional working shape medication adherence following MI. Addressing these gaps is an important step towards informing a more targeted, tailored, collaborative, and sustainable approach to supporting adherence in this population.

### Aim

This study aims to explore HCPs’ perspectives on barriers and facilitators to medication adherence post-MI and identify the key HCP behavioural determinants influencing medication management within hospital and primary care settings.

## 2. Materials and Methods

### 2.1. Governance

Ethics approval to conduct this study was granted by the Social Research Ethics Committee, University College Cork, Cork, Ireland, Log No: 2024-163. All participants were emailed to the participant information sheet and consent form prior to the interview. Written informed consent was obtained in advance, and verbal consent was reconfirmed at the start of each interview. This study was reported in line with the Consolidated Criteria for Reporting Qualitative Studies (COREQ) guidelines (Supplementary File S1) [22]. No incentives were offered to participants.

### 2.2. Study Design

A qualitative descriptive study design using semi-structured interviews was used to explore HCPs’ perspectives on barriers and facilitators to medication adherence following MI. This design was chosen to generate rich, contextualised insights into professional practices and experiences, without imposing prior hypotheses [23]. The study design prioritised flexibility and participant preference, with both online video (via MS-Teams, version 26005.204.4249.1621.) and in-person interviews offered to accommodate individual needs. This approach was considered most suitable for facilitating open and comfortable dialogue while ensuring broad participation.

### 2.3. Theoretical Domains Framework (TDF)

The Theoretical Domains Framework (TDF) is a comprehensive, validated framework that synthesizes 84 theoretical constructs from 33 behaviour change theories, organized into 14 domains: Knowledge; Skills; Social/Professional Role and Identity; Beliefs About Capabilities; Optimism; Beliefs About Consequences; Reinforcement; Intentions; Goals; Memory, Attention and Decision; Environmental Context and Resources; Social Influences; Emotion; and Behavioural Regulation [24,25]. The TDF has been widely applied in implementation research to systematically explore the cognitive, social, and environmental factors that influence clinical practice [26]. It has been particularly useful in identifying barriers and facilitators to a range of health-related behaviours across diverse healthcare settings and professional groups, such as stroke survivors [27]. In this study, supporting medication adherence post-MI was conceptualised as HCPs’ behaviour involving counselling, patient education, prescribing decisions, and coordination of follow-up care, taking into consideration the wide experience of HCPs. The TDF was therefore selected as the analytical framework because it provides a comprehensive, theory-informed structure for examining the cognitive, social, and environmental determinants of such clinical behaviours.

### 2.4. Eligibility Criteria

HCPs were eligible to participate in this study if they were involved in the care of people following an MI. Eligible professionals included GPs, cardiologists, nurses, and pharmacists with a minimum of one year’s experience in cardiovascular care.

### 2.5. Participant Sampling

Participants were recruited in the southwest region of Ireland using a combination of purposive, convenience, and snowball sampling methods. To identify individuals interested in participating, a standardised email containing an information leaflet and consent form was circulated to relevant professional networks. A purposive maximum variation sampling approach was used to ensure that a diverse range of perspectives relevant to post-MI medication management were represented [28]. To achieve this, the study aimed to recruit at least two participants from each professional group involved in medication management (pharmacists, general practitioners, cardiologists, and nurses). In addition, representation across practice locations (urban/rural) and settings (public, private, and mixed) was sought to capture variation in organisational context. Variation in years of experience was also considered to ensure inclusion of both early-career and senior clinicians, with a minimum requirement of one year of experience to ensure adequate familiarity with post-MI care pathways. These sampling targets were intended to support breadth and depth of insight rather than to achieve numerical proportionality, consistent with qualitative research principles [28]. Invitations were distributed via professional networks, including professional organisations (e.g., Irish College of General Practitioners) and relevant specialist groups. In addition, professional contacts of both researchers and participants were used to facilitate snowballing, ensuring variation in discipline and care setting.

In qualitative research, sample adequacy is guided by the depth and relevance of the data rather than statistical calculation, and contemporary qualitative guidance acknowledges that while saturation remains a widely used indicator, it is also a concept that attracts ongoing debate regarding its interpretation and application [29]. Nevertheless, established guidelines suggest that thematic saturation is commonly achieved within 12–20 interviews, depending on the diversity of participants [30,31]. The sample size was further justified by the principle of information power, which posits that studies with a narrow aim, strong theoretical grounding, high-quality dialogue, and participants with relevant, specific experience require fewer participants to yield meaningful insights [32]. In this study, the sampling matrix ensured that perspectives from all key subcategories of healthcare professionals involved in post-MI medication management were represented, providing a strong foundation for assessing the completeness of the emerging analysis. Data collection continued until no new themes were generated, defined as the point at which additional interviews no longer yielded novel insights relevant to the research questions [33,34,35]. Sample adequacy was considered reached after the ninth interview, and three further interviews were conducted to confirm the stability and completeness of the thematic structure, thereby strengthening confidence in the robustness and trustworthiness of the findings.

### 2.6. Data Generation

Semi-structured interviews were conducted by FEK using an indicative topic guide (Supplementary File S2), which was developed based on the TDF [24]. Findings from a recent qualitative systematic review [4] and discussions within the research team informed the development and refinement of the guide to ensure relevance, clarity, and comprehensive coverage of key behavioural domains related to medication adherence post-MI [25]. This approach ensured that the guide was theoretically grounded, comprehensive, and clearly structured, while remaining flexible and relevant to the local clinical context and participants’ experiences of supporting medication adherence post-MI. Participant demographics were collected using a data collection form completed before the interview (Supplementary File S3). All interviews were recorded on MS Teams, transcribed verbatim, and anonymised through redaction prior to analysis.

### 2.7. Data Analysis

#### 2.7.1. Analytic Process

All transcripts were uploaded to NVivo 12 (QSR International Pty Ltd., Doncaster, Australia, Version 12) to support data management and facilitate analysis. In Phase 1, transcripts were read and re-read by FEK to ensure immersion. Phase 2 involved conventional content analysis, with open coding used to inductively generate preliminary codes and capture early thematic patterns without imposing a hierarchy. In Phase 3, directed qualitative content analysis, following the method outlined by Hsieh and Shannon, was applied [36]. An initial coding framework, structured around the 14 TDF domains, was developed, with the theoretical definitions and component constructs for each domain guiding the process. This allowed the findings to be systematically mapped onto the relevant domains of the TDF, ensuring that both emergent themes and theory-driven constructs were captured. To strengthen rigour, a second researcher (LS) reviewed a random subset of transcripts (25%, n = 3), applied independent coding, and mapped emerging themes onto TDF. Final coding decisions were reached through consensus discussions, which weighed three considerations when determining the most dominant domains: (i) how frequently particular beliefs appeared, (ii) the extent of variation or contradiction in those beliefs, and (iii) the perceived influence of those beliefs on how HCPs support medication adherence post-MI.

#### 2.7.2. Reflexivity: Researcher Backgrounds

The authors, all pharmacists, adopted a reflexive stance throughout the research process, acknowledging how their professional backgrounds and experiences could influence data interpretation, particularly regarding the roles of pharmacists and other healthcare professionals in supporting medication adherence. To mitigate potential bias, in addition to the independent double-coding of some transcripts, regular team discussions were held to critically examine assumptions and interpretations.

## 3. Results

### 3.1. Participant Recruitment

Twelve interviews were conducted between December 2024 and May 2025 with HCPs practicing in the southwest region of Ireland. Participant demographics are detailed in Table 1. The sample included four pharmacists, two GPs, three cardiologists, and three nurses. The mean interview duration was 41 min, with a range of 30 to 50 min.

### 3.2. Key Determinants Influencing Medication Adherence Post-MI from HCPs’ Perspective

The analysis identified 15 facilitators and 13 barriers to medication adherence, and seven themes that could act as barriers, facilitators, or both, and were mapped across 10 TDF domains (Table 2 and Table 3). These predominant domains are outlined below, accompanied by conventional themes and supported by illustrative quotations. Due to the multidimensional nature of participants’ responses, some quotations may align with more than one TDF domain.

#### 3.2.1. TDF Domain: Social/Professional Role and Identity

- Interdisciplinary collaboration

All HCPs emphasised the importance of multidisciplinary collaboration, particularly amongst nurses, cardiologists, GPs, and pharmacists, in optimising patient outcomes following MI. In hospital contexts, nurses were reported as critical in the identification of potential problems during discharge planning. Nurses were frequently described as having the time to identify potential adherence issues, particularly in outpatient settings such as primary care clinics or structured cardiac rehabilitation sessions, where they often spend longer with patients than physicians.

Some HCPs highlighted the ideal scenario as a cooperative process in which all professionals consistently reinforce the importance of medication adherence. However, it was acknowledged that this level of communication was not always achieved in practice, and gaps in coordination could pose significant challenges.

“Even though the pharmacist gives the session, the cardiac rehab nurse would always be there at the same time. So, we would […] work with each other just in terms of if there was anything that I noticed in that session, was there any questions that came up, I would […] go over to the nurse at the end and say, look, Mr. XYZ was asking me all the questions about this. Maybe could you follow up with him in a few weeks and see […] how he’s getting on? And let me know, for example. So sometimes it’s just that understanding, […], the nurse is going to have more frequent contact with this person. But at the same time, medication probably obviously is more of my kind of remit.”

(P7, Hospital Pharmacist)

“Well, it should be to lead a team of professionals who are all singing from the same hymn sheet, but sadly, it’s not always like that […] staff can have a complement, which they should be trying to achieve. It’s often underprovided, and then they’re rushing from one person to the next […] And of course, if everyone is not listening and partaking in the discussion, the patient loses out. It’s not us necessarily.”

(P11, Cardiologist)

“It’s […] having a structured programme, and as we are lucky enough to have in cardiology with cardiac rehabilitation, I think that is the key to making sure that patients get a holistic approach to their care and everybody is giving the same message, albeit from a slightly different angle.”

(P10, GP)

b. Tailored approaches and strategies (holistic care)

HCPs emphasized the importance of holistic, patient-centred care and tailoring adherence support to individual needs following MI. While core information provided to patients was generally consistent, participants described adapting their approach according to each patient’s preferences, educational background, lifestyle, and specific barriers. They stressed the value of working collaboratively with patients to develop personalised strategies for medication use and to manage potential side effects. Pharmacists and nurses expressed a sense of responsibility in helping patients establish regular routines and providing ongoing support. Practical examples included simplifying regimens to reduce pill burden, aligning dosing schedules with daily activities such as mealtimes, offering additional counselling to those with lower health literacy, and recommending tools like smartphone reminders or mobile health apps.

“I do think it’s very individual, right? So, I can recommend something to one patient, and it can suit them. And you could recommend something to another patient that they could say, I don’t like putting reminders on my phone, for example. Or one man I remember one day doesn’t have a mobile phone. He only had his landline, so I think reminders on a phone or an alarm clock, or, you know, just a watch would be one kind of basic way.”

(P7, Hospital Pharmacist)

“Leave your morning medications out with a breakfast cereal or with the breakfast bowls or whatever. Leave something […] with your toothpaste. Whatever it is, just to […] create a link at the start until they get into the habit. And then after that, if they’re finding it difficult devices like dosette boxes or blister packs”

(P9, Hospital Pharmacist)

c. Continuity of care: Chronic Disease Management

HCPs highlighted the value of the Chronic Disease Management programme in supporting medication adherence following MI. They viewed Chronic Disease Management as a beneficial framework that facilitates continuity of care and provides structured opportunities for follow-up with patients. HCPs expressed appreciation for having adequate time during Chronic Disease Management consultations and noted that the programme allows for at least biannual patient interactions. However, they also acknowledged a key limitation: not all patients are eligible for enrolment in the Chronic Disease Management, which restricts the programme’s reach and its potential impact on long-term medication adherence. In Ireland, the Chronic Disease Management treatment programme is accessible only to those in receipt of publicly funded healthcare [10].

“I think the goal is obviously to have everybody adhere to medications that they need that they can tolerate, and Chronic Disease Management, I think, has been the best measure that has come into general practice in recent times to allow us to kind of realise that goal.”

(P4, GP)

#### 3.2.2. TDF Domain: Environmental Context and Resources

a. Lack of resources

HCPs expressed significant concerns about the constraints impacting their ability to provide optimal post-MI care. HCPs noted that standard 15 min consultation slots are insufficient to address the complex needs of these patients, particularly when discussing medication adherence and long-term management. The strain on the healthcare system due to shortages of GPs, hospital doctors, and pharmacists was seen as a significant barrier to the delivery of comprehensive care. This lack of resources limited the time spent with patients and affected the quality of follow-up and continuity of care. A hospital pharmacist observed that patients were often reluctant to ask questions about their medications, which may lead to a lack of understanding of their importance and, in turn, affect adherence.

“A lot of times, patients can be afraid to ask the consultant [cardiologist] because, […] they’re seen as terribly busy people. They’re coming in and out of the room very quickly, and they [patients] don’t like bothering them [consultant] too much. So sometimes that can be a barrier”

(P9, Hospital Pharmacist)

“I suppose the measure of adherence we would typically use is ringing the community pharmacy and asking them if the patient gets this dispensed? When was their last dispensing? That’s obviously only giving us adherence to the level of collecting a prescription or getting it dispensed. And then talking to the patient themselves in terms of whether they actually take it.”

(P7, Hospital Pharmacist)

Collectively, these reflections underscore how resource limitations hinder the delivery of the individualised care necessary to support medication adherence post-MI. Participants suggested that enhancing digital solutions or “digitalising the prescription process” could help streamline workflows and reduce administrative burden. Specifically, this referred to the expansion of electronic prescribing systems that allow prescriptions to be generated, transmitted, and renewed digitally between GPs, pharmacies, and hospitals. Participants reported that electronic prescribing could reduce errors, improve efficiency, and free up time for more patient-focused care. These insights reflected a healthcare system strained by limited resources, where both human and technological enhancements are needed to improve care quality and support post-MI medication adherence.

“It is really crazy in 2025 that we have systems that can’t talk to each other and if we had systems that speak to each other, the workload would be much less for everybody and we’d be able to be far more efficient with our time and spend time actually educating patients rather than spending time phoning the pharmacy to find out what medications patients [are] actually taking because the patient doesn’t know.”

(P10, Cardiologist)

b. Adherence support tools

HCPs noted that practical aids such as dosette boxes, blister packs, and medication alarms can be effective in supporting some patients with post-MI medication adherence. HCPs believe that these tools help to simplify complex regimens, serve as visual or auditory reminders, and promote routine, particularly for patients who struggle with memory or organisation. Whilst not universally effective, they were reported as valuable components of a broader strategy to support consistent medication use.

“But I’m a big fan of the blister packs. They’re really, really helpful. And then there’s no risk of doubling up, either, because the days are outlined. So, there’s no, there’s no risk that you’re saying, oh, did I take my medicines? One clearly sees they did.”

(P1, Nurse)

#### 3.2.3. TDF Domain: Knowledge

a. Patients’ understanding and education

All HCPs emphasised the importance of reinforcing medication adherence and explaining to patients the purpose of each medication prescribed post-MI. HCPs noted that while some patients were eager to understand the details, others preferred only a basic overview of the information. HCPs reported adapting their communication strategies and content accordingly, with some focusing on specific topics, e.g., side-effects, while others concentrated solely on the indication and benefits. Regardless of the approach, all reported the importance of encouraging patients to report any unusual symptoms. To enhance understanding and reduce anxiety, HCPs consistently reassured patients about the safety of their medicines and used simple, clear, and non-technical language to aid comprehension.

“One of my policies would have been always, if anybody is starting a new medication, you need to take the opportunity to have a word with them to make sure that they know what they’re taking.”

(P2, Community Pharmacist)

b. Health literacy

HCPs expressed concern about patients’ limited understanding of their medications and the implications for long-term adherence post-MI. Several HCPs observed that patients often mistakenly view their treatment as short-term, like a course of antibiotics, and were unaware that these medications are intended for lifelong use. Poor communication and inadequate information at the point of hospital discharge were reported as contributors to this confusion. HCPs emphasised that, in their view, when patients do not understand why they are taking medicine, they are less likely to take it consistently. This nonadherence is then compounded should they experience undesirable effects, which they likely attribute to medicine. One participant noted that patients often retain only a small fraction of the information provided, and may misunderstand a significant portion of it, further complicating adherence. These insights underscore the importance of enhancing health literacy through clear and consistent communication, as well as patient education, to facilitate effective long-term medication use.

“I think the problem is if they don’t know why they’re taking medication, they’re not going to take it. They’re naturally going to be slightly uncertain, and if they feel peculiar, they’re going to sort of blame it on the medication.”

(P11, Cardiologist)

“You do meet people that will be coming in through the Emergency Department who haven’t been taking their tablets for years because they thought it was a short-term strategy.”

(P12, Cardiologist)

“Patients remember 10% of what we say to them. They don’t remember much more than that. And they get 50% of that 10% wrong, so we’re on a hiding to nothing”

(P12, Cardiologist)

#### 3.2.4. TDF Domain: Emotion

a. Psychological barriers to medication use

Following an MI, HCPs believed many patients experienced heightened anxiety and emotional distress, which can be an additional barrier to medication adherence. The fear of being chronically ill, or the overwhelming thought of having to take multiple medications for life, can lead to avoidance, denial, or inconsistent use, in the opinion of HCPs. Some individuals may associate medications with their illness, reinforcing a negative emotional response and resistance to treatment.

“I just see anxiety around being on medication. […] some people see it as a failing. Some people see it as, you know, a sign of ageing, which they don’t want to be. And you know, so acceptance of the condition, and again with MI, like you’re dealing with a huge mortality scare.”

(P1, Nurse)

b. Fear as a motivator

Conversely, HCPs noted that fear could also serve as a powerful motivator of adherence. Many patients report to HCPs on how surviving a life-threatening event like a heart attack serves as a wake-up call, prompting strict compliance with their prescribed medication regimen. The emotional impact of nearly dying often leads to a strong desire to prevent another cardiac event, encouraging individuals to take their medications as a means of regaining control and prolonging life, having been given a second chance.

“I think people have got such a fright having had a heart attack that they’re like they want to be compliant with their medicines.”

(P5, Nurse)

“Initially, you would find the first two to three months post the MI, they’re scared, you know, they feel like they’re lucky to be alive, that they’ve survived this, and they’re their best person ever. They’ve given up smoking; they’re eating well, they’re exercising. They’re losing weight. They’re taking their medicines, and that’s all for the initial period.”

(P6, Nurse)

#### 3.2.5. Social Influence

a. Family involvement

HCPs emphasized that family support plays a critical role in promoting medication adherence, especially among older adults or those with cognitive impairment. Caregivers and family members step in to assist with organising and administering medications, attending medical appointments, and reinforcing the importance of adherence. Their involvement can significantly enhance the patient’s ability to consistently and accurately use prescribed therapies. Some HCPs used motivational strategies tailored to personal goals, such as encouraging patients by referencing meaningful life events to foster a sense of purpose around medication adherence.

“You’ve said that you want to walk your daughter down the aisle on her wedding day. Well, you take your antihypertensives and your aspirin. That’s your part to play in, ensuring that you, you know, achieve your objectives, be it to live a long, healthy retirement. What I find a lot of them say, well, actually, I want to walk my daughter down the aisle or I want […] to be at my grandson’s communion.”

(P3, GP)

“In the majority of cases, in my view, once there’s a fairly decent family structure in place, it generally works. If you have a partner who’s encouraging the other partner to take the medication, most of those situations are generally fine. What you do find is people living on their own, and especially as they get older, compliance can be a problem”

(P2, Community pharmacist)

b. Overreliance on family members

Excessive dependence on family members can sometimes lead to gaps in understanding and, hence, in the execution of medication-related tasks. When patients rely entirely on others, they may miss key information about their medications, such as dosage changes, potential side effects, and the rationale behind each prescription. Additionally, if family members are not fully informed or trained, they may unintentionally provide incorrect information, leading to missed doses or improper administration. This highlights the importance of ensuring both patients and caregivers receive adequate education and engagement from healthcare professionals.

“The interaction with the patient is reduced because a representative is coming for them [the medicines]. And so that’s a negative because you’re not empowering the patient with knowledge about what they’re taking. You’re just trying to make sure that they’re taking it safely and correctly as a baseline. But unfortunately, I think then it’s probably at the cost of the education piece.”

(P8, Community pharmacist)

## 4. Discussion

This study explored HCPs’ perspectives on factors influencing post-MI medication adherence and highlighted key behavioural determinants shaping medication management across hospital, primary care, and community pharmacy settings. While many findings reinforce existing literature on adherence [37,38,39,40] this study makes two significant contributions. First, it demonstrates how HCPs’ real-world experiences contextualise these barriers and facilitators in the unique post-MI setting, where sudden transitions, polypharmacy, and emotional vulnerability differentiate people post-MI from those with other chronic conditions. Second, it identifies opportunities for practice improvements and policy changes by clarifying where HCPs feel most constrained and where their input can directly shape effective interventions. Taken together, these contributions enhance understanding not only by reaffirming existing knowledge, but also by embedding it within a real-world, multidisciplinary post-MI care pathway.

To ensure these findings were robust and reflective of diverse clinical perspectives, the final sample included HCPs from multiple professional backgrounds, including community pharmacists, hospital pharmacists, GPs, and nurses. The sample size was further justified using the principle of information power. Accordingly, a focused study aim, a theoretically informed interview guide, and participants with direct post-MI medication management experience supported the use of a smaller, information-rich sample [32]. Consequently, the inclusion of twelve participants was considered sufficient to address the research aim and generate meaningful, in-depth insights into the behavioural determinants influencing medication adherence post-MI. Therefore, the strength of the findings lies not in numerical breadth but in the depth and relevance of multidisciplinary experiential knowledge captured.

Crucially, these insights must also be interpreted within the broader organisational context of post-MI care in Ireland. Following hospital discharge, patients transition from specialist cardiology services to GP-led primary care, typically supported by nursing and allied health professionals. Within this system, ongoing communication between primary care and community pharmacy enabled challenges, such as medication adherence difficulties, to be identified, shared, and addressed collaboratively. As such, the organisational structure not only shaped HCPs’ experiences but also influenced perceived barriers and facilitators, reinforcing the practical relevance and applicability of the identified behavioural determinants.

Our findings align with prior work showing that patient beliefs about necessity and concerns are central to medication adherence behaviours [41,42,43,44]. Patients’ perceptions of medication emerged as central determinants of adherence, echoing extensive research demonstrating that patients’ necessity beliefs and concerns strongly shape adherence behaviours [45,46]. Consistent with prior work, HCPs reported that fear of recurrence and trust in prescribed therapies acted as powerful motivators, while misconceptions, such as viewing medications as short-term treatments or concerns about dependency and side effects, undermined adherence and acted as barriers [47,48,49,50]. These observations are consistent with recent qualitative evidence from both patients and nurses following acute coronary syndrome, which highlighted the central role of perceived risk, benefits, self-efficacy, and cues to action in shaping adherence behaviours across care settings [51]. This underscores the importance of timing and staging education, a consideration often underemphasized in the adherence literature [52,53]. These findings suggest that adherence interventions may be most effective when time-sequenced, with intensive support early after discharge and structured reinforcement as fear subsides.

HCPs frequently reported that patients struggled to understand the lifelong nature of post-MI treatment and retained limited information provided at hospital discharge. This finding mirrors well-documented challenges associated with care transitions, where information overload and emotional distress impair retention [54]. Evidence supports strategies such as staged education, reinforcement during follow-up visits, pharmacist-led medication reviews, and written or digital summaries to improve understanding and long-term adherence [55]. Poor health literacy and ineffective communication were persistent barriers, a finding which aligns with prior studies showing that inadequate health literacy is associated with non-adherence and adverse outcomes [56,57]. While efforts to simplify language, reduce jargon, and use visual or mnemonic aids were described, misunderstandings often persisted. This points to the need for iterative, reinforced communication and possibly structured literacy-sensitive interventions, particularly at key transition points such as hospital discharge. Our results also echo reports from Slovenia and the United States, where continuity-of-care gaps between hospital and community providers undermined medication use [58,59]. While education remains the dominant strategy used by HCPs, behavioural science research shows that knowledge alone seldom translates into sustained behaviour change; it needs to be a developing habit [60].

Although HCPs in this study perceived adherence aids such as blister packs and pill organisers as being helpful tools to support routine medication-taking, the broader evidence base on their effectiveness is mixed. A systematic review of electronic multi-compartment medication devices with reminder systems found that the existing studies were small, methodologically limited, and highly heterogeneous, providing insufficient high-quality evidence to draw firm conclusions about their impact on adherence across populations and settings [61]. Additionally, a randomised clinical trial examining low-cost reminder devices, including standard pillboxes and digital timer caps, did not demonstrate significant improvements in adherence compared with usual care among people taking multiple chronic medications, suggesting that simple reminder aids alone may be insufficient to change behaviour without additional supportive strategies [62]. These findings highlight that while adherence aids remain a commonly recommended component of adherence support, their real-world effectiveness may depend on factors such as patient engagement, regimen complexity, and integration into broader behavioural and educational interventions. In parallel, emerging digital health research suggests that technology-enabled support may help address some of these behavioural and contextual challenges. A recent mixed-methods study of remote patient management following MI identified distinct adherence personas, demonstrating that engagement with digital interventions is shaped by patients’ beliefs, motivation, and self-management capacity [63].

Several barriers reported by participants were classified within TDF domains such as ‘Environmental Context and Resources’ and ‘Social Influences’. Guided by the TDF, the findings identify key behavioural determinants and highlight opportunities to embed evidence-based behaviour change techniques into routine practice [16,25,64]. Strengthening multidisciplinary collaboration, enhancing communication, and addressing system-level barriers are therefore crucial to empowering patients to effectively manage their medications over time, ultimately improving long-term adherence and reducing recurrent cardiovascular events. Some findings merit particular consideration as they challenge prevailing assumptions in the adherence literature. HCPs in this study described family involvement as having a dual role in medication adherence, acting as both a facilitator and, in some cases, a barrier. While family support often reinforced routines and reminders, over-delegating responsibility to family members was perceived as reducing patient engagement. These findings suggest that family involvement may be most beneficial when roles are clearly defined. Shared patient–caregiver education, explicit clarification of responsibilities, and inclusion of family members in medication counselling may help maximise supportive effects while minimising unintended consequences [65,66,67]. Additionally, medication cost did not emerge as a perceived barrier, contrasting with prior evidence from the United States of America showing that full financial coverage improves adherence following MI [2]. This difference may reflect the Irish healthcare context, where means-tested schemes ensure that patients pay a minor co-payment (€0.50 per medication) or a capped monthly cost (€80.00) in 2025 for their medications [68].

These findings support shifting person-centred, theory-informed interventions that embed behaviour change techniques, such as simplifying regimens, goal setting, using cues and reminders, and shared decision-making, into routine care. However, participants’ accounts also point to critical system-level constraints that limit the consistent delivery of such interventions. Moreover, HCPs repeatedly emphasised the importance of follow-up and continuity, suggesting that structured programmes like the Irish Chronic Disease Management programme could be strengthened through systematic medication reviews, scheduled check-ins, or digital adherence support. Realising these opportunities, however, requires supportive infrastructure at the health system level.

A key implication for Irish policymakers is the absence of a national, interoperable digital health system in Ireland that connects primary care settings (pharmacies and GP practices) or primary and secondary care. Participants prioritised improvements in communication and the lack of access to shared medication information, for example, patients’ files, as significant barriers to adherence support. Rather than requesting entirely novel digital innovations, healthcare professionals expressed a preference for functional digital solutions, such as shared prescribing and dispensing records, and streamlined follow-up processes. The lack of a comprehensive electronic health record system across Irish public hospitals remains a significant barrier to coordinated post-MI care. In the absence of integrated digital records, critical information such as medication changes, discharge instructions, and follow-up plans may not be consistently or promptly communicated to primary care providers. This discontinuity at hospital discharge undermines opportunities for early adherence support. Strengthening digital health infrastructure is therefore a key system-level priority to improve information transfer, prescribing safety, and the delivery of secondary prevention in routine practice.

The findings of this study also provide clear direction for the development of practical, theory-informed behaviour change interventions. Barriers identified within the TDF domains of Knowledge and Memory, Attention and Decision Processes suggest the need for staged education delivered across care transitions, reinforced through follow-up consultations and pharmacist-led medication reviews. Determinants within Social Influences highlight opportunities to integrate structured patient–family education that clarifies roles and promotes shared responsibility without undermining patient autonomy. Barriers related to Environmental Context and Resources indicate that interventions should focus on simplifying medication regimens and schedules, embedding adherence checks into routine reviews, and leveraging existing structured programmes such as Cardiac Rehabilitation and Chronic Disease Management. Finally, the prominence of Beliefs about Consequences and Motivation suggests that early fear-driven adherence may be supported by timely reinforcement, goal setting, and habit-formation strategies, including reminders and cues, to sustain adherence as motivation wanes over time. These examples demonstrate how the identified behavioural determinants can be operationalised into feasible, practice-based interventions across the post-MI care pathway.

### Limitations

All participants were based in the southwest region of Ireland, which may limit the generalizability of the findings to other locations with different healthcare systems, resources, or post-MI care models. Although the sample included a range of healthcare professionals, including GPs, nurses, pharmacists, and cardiologists, broader perspectives, such as those from other regions or healthcare roles, such as an occupational therapist, may have offered additional insights. The use of purposive sampling may have introduced a degree of selection bias, limiting the variation amongst participants. As such, the findings reflect the views of the professionals interviewed and should be interpreted with caution when applied to wider populations. The research team’s shared professional background in pharmacy may have shaped interpretive perspectives, and this potential bias was addressed through reflexive practice and joint review of coding and theme development.

## 5. Conclusions

This study provides novel insights into post-MI medication adherence by examining this complex issue through the perspectives of healthcare professionals across multiple disciplines. Beyond reaffirming known adherence challenges, it demonstrates how HCPs’ real-world experiences contextualise these barriers and facilitators within the unique post-MI setting. Importantly, the findings identify specific points in the care pathway where HCPs feel most constrained and where their expertise could meaningfully inform the design of targeted, practice-ready interventions. By applying behaviour change theory to identify opportunities to strengthen continuity of care, align education with patient capacity, and support personalised decision-making, this study lays a foundation for developing HCP-driven, behaviourally informed strategies to enhance post-MI medication adherence.

Future work will map the identified TDF domains onto the capability, opportunity, and motivation—behaviour (COM-B) model to more directly inform the design of behaviour change interventions in this setting, following approaches used in similar qualitative studies [69]. Using the COM-B model, preliminary insights indicate that patients’ capability may be affected by factors such as health literacy and cognitive load, opportunity may be shaped by system-level coordination and access to follow-up support, and beliefs about treatment necessity, side effects, or medication burden may influence motivation. Mapping these determinants onto the Behaviour Change Wheel (BCW) can inform the next stage of research, identifying appropriate intervention functions, such as education, training, enablement, and environmental restructuring, along with policy- or service-level strategies to strengthen continuity of care [69]. These findings will directly guide the co-design of a targeted, theory-informed medication adherence intervention for post-MI patients [24,69].

## Figures and Tables

**Table 1:** Participant demographics.

Healthcare Professionals’ Characteristics		Number of Healthcare Professionals Interviewed	Minimum Number of Healthcare Professionals Required in the Sampling Strategy
Sex	Male	4	-
	Female	8	-
Discipline	Community Pharmacists	2	2
	Hospital Pharmacists	2	2
	General practitioners	2	2
	Cardiologists	3	2
	Nurses	3	2
Years in practice	0–9	3	1
	10–19	1	1
	≥20	8	1
Practice location	Urban suburb	8	1
	Rural/semi-rural	1	1
	City centre	3	1
Setting	Public	6	2
	Private	4	2
	Mixed (Public and Private)	2	2

**Table 2:** Barriers to Post-Myocardial Infarction Medication Adherence: Healthcare Professionals’ Perspectives across Key Theoretical Domains Framework Domains.

TDF Domain	Key Theme
Social/professional role & identity	Co-morbidities
	Challenges in self-administering medication post-discharge
	Differences between patients (age/sex)
	Interdisciplinary collaboration
Environmental context and resources	Lack of resources
	Medication burden
	Technology
	Time & timing
	Pandemic effect
	Medication accessibility
	Language (medical terms)
	Lifestyle-related factors
Knowledge	Healthcare professionals’ knowledge
	Health literacy
Emotion	Psychological factors in medication use
Social Influence	Overreliance on family members
Beliefs about consequences	Myocardial infarction (and/or) medication adherence
	Polypharmacy
Memory, attention & decision	Cognitive impairment
	Intentional non-adherence

**Table 3:** Facilitators to Post-Myocardial Infarction Medication Adherence: Healthcare Professionals’ Perspectives across Key Theoretical Domains Framework Domains.

TDF Domain	Key Theme
Social/professional role & identity	Continuity of care, as in the chronic disease management programme
	Healthcare professionals’ impact
	Differences between patients (age/sex)
	Tailored approaches and strategies (holistic care)
	Interdisciplinary collaboration
Environmental context and resources	Technology
	Time & timing
	Provision of written information
	Medication accessibility
	Lifestyle-related factors
	Adherence support tools
Knowledge	Patient education and understanding
	Healthcare professionals’ knowledge
Emotion	Fear as a motivator
	Empathy and support
Social influence	Family Involvement
	Patients & healthcare professionals’ relationship (trust in healthcare professionals and peer support)
	Patient engagement
Beliefs about consequences	Reinforcing adherence through consequences
Behavioural influence	Behavioural regulation
Beliefs about capabilities	Patient empowerment & shared decision-making
Goals	Healthcare professionals’ goals

## Data Availability

The data presented in this study are available upon reasonable request, as sharing could compromise participant anonymity given the single-site study design. This restriction was a condition of the ethics approval and is consistent with the information provided to participants in the study information leaflet and consent form. Further inquiries can be directed to Professor Laura J. Sahm.

## Supplementary Materials

The following supporting information can be downloaded at: https://www.mdpi.com/article/10.3390/pharmacy14010023/s1. File S1: COREQ Checklist; File S2: Theoretical Domain Framework-informed interview topic guide; File S3: Healthcare professionals’ demographic information for the interview study. References [70,71] have been mentioned in the text.

## Abbreviations

The following abbreviations are used in this manuscript:

MI	Myocardial Infarction
HCPs	Healthcare professionals
GP	General Practitioners
TDF	Theoretical Domains Framework
WHO	World Health Organisation
HSE	Health Service Executive
SDM	Shared decision-making
COREQ	Consolidated Criteria for Reporting Qualitative
COM-B	Capability, Opportunity, and Motivation-Behaviour
BCW	Behaviour Change Wheel
