# Supplementary material for: Healthcare Professionals’ Perspectives on Barriers and Facilitators to Medication Adherence Post Myocardial Infarction: A Qualitative Study Using the Theoretical Domains Framework
Source: Pharmacy (Basel). 2026 Feb 2;14(1):23. doi: 10.3390/pharmacy14010023 (PMC12921787; doi:10.3390/pharmacy14010023)
Supplement: Supplementary file 1 [file pharmacy-14-00023-s001.zip › Supplementary file _3 demographics.pdf]

Healthcare professionals' demographic information for the interview study

Interview Number:

Date:

Demographic Information

Gender:

Age:

18-29 ☐ 30-39 ☐ 40-49 ☐ ≥50 ☐

Discipline: ....

Years of experience:

0-4 ☐ 5-9 ☐ 10-19 ☐ ≥20 ☐

Details pertaining to practice

Practice location:

City centre ☐ Urban suburb ☐ Rural/Semi-rural ☐

Working in:

Community ☐ Hospital ☐
