# Supplementary material for: Healthcare Professionals’ Perspectives on Barriers and Facilitators to Medication Adherence Post Myocardial Infarction: A Qualitative Study Using the Theoretical Domains Framework
Source: Pharmacy (Basel). 2026 Feb 2;14(1):23. doi: 10.3390/pharmacy14010023 (PMC12921787; doi:10.3390/pharmacy14010023)
Supplement: Supplementary file 1 [file pharmacy-14-00023-s001.zip › Supplementary File_2 Topic guide.pdf]

Supplementary File S2. Theoretical Domain Framework-informed interview topic guide to examine healthcare professionals' perspectives on barriers and facilitators to medication adherence following myocardial infarction.

Area	Interview question
General questions	When do you first contact patients with MI[1]? AND what do you think are the common barriers to medication adherence post-MI patients in your practice?
	How often do you encounter issues with adherence post-MI and at what stage e.g. (initiation, implementation or persistence)
Knowledge	Do you think patients receive adequate information regarding their medical condition and medication post-discharge? AND how do you educate the patients about their medication post-MI?
	Do you use specific tools or materials to help patients understand their treatment plan?
Memory, Attention, Decision Processes	How do you think we can ensure that post-MI patients remember to take and refill their medications as prescribed?
	What decision-making strategies do you think will help the patients to face challenges in medication adherence?
Social Professional Role & Identity	How do you perceive your role in supporting patients facing challenges with medication adherence?
	How do you collaborate with other HCPs[2] to ensure medication adherence in post-MI patients?
Beliefs about Capabilities	How confident are you with your ability to effectively communicate with the patients regarding the importance of medication adherence post-MI? And can you mention an example when you successfully helped a patient to adhere to their medication?
	How do you involve patients in decision-making about their medications to enhance adherence?

[1] MI: Myocardial Infarction.
[2] HCPs: Healthcare Professionals.

Beliefs about consequences	How do you mention the short- and long-term benefits of medication adherence to the patients? And do you mention the consequences of non-adherence as well?
Optimism	How optimistic are you about medication adherence improvement post-MI? What strategies do you use to follow up with patients who might struggle with adherence?
Intentions	How do you assess patients' intentions to adhere to their medication regimen?
Goals	What are your goals regarding improving medication adherence among MI patients? And how do you prioritise these goals within practice?
	Do you tailor your approach based on the individual needs and preferences of patients?
Reinforcement	Can you share some examples of how you address barriers to medication adherence and reinforce positive behaviour?
Emotion	How do you address emotional factors, such as anxiety or depression, that may affect medication adherence in post-MI?
Social influence	Do you involve carers or family members in supporting medication adherence? Can you share some examples?
Environmental context & resources	Do lack of time and resources affect the information given to the patients regarding the medication prescribed?
	How do you address the factors that can act as a barrier for medication adherence, for example accessibility or cost?
Skills	How do you educate your patients regarding medication administration techniques? And how do you assess their understanding and ability to apply those techniques?
Behavioural regulation	Do you assist patients to establish routines for their medication adherence to support consistent medication adherence?
