# Supplementary material for: Healthcare Professionals’ Perspectives on Barriers and Facilitators to Medication Adherence Post Myocardial Infarction: A Qualitative Study Using the Theoretical Domains Framework
Source: Pharmacy (Basel). 2026 Feb 2;14(1):23. doi: 10.3390/pharmacy14010023 (PMC12921787; doi:10.3390/pharmacy14010023)
Supplement: Supplementary file 1 [file pharmacy-14-00023-s001.zip › supplementary_file_ 1 COREQ Checklist.pdf]

Supplementary File S1: COREQ Checklist [1]

**Domain 1: Research team and reflexivity**			
**Personal characteristics**			
**1.**	Interviewer/facilitator	Which author/s conducted the interview or focus group?	FEK conducted the interviews.
**2.**	Credentials	What were the researcher's credentials (e.g. PhD, MD)?	At the time of undertaking the interviews, FEK's credentials were a BSc in Pharmacy and Member of Pharmaceutical Society in Egypt (MPSE).
**3.**	Occupation	What was their occupation at the time of the study?	FEK is an Egyptian-registered pharmacist who was undertaking a PhD in clinical pharmacy research in Ireland at the time this study was conducted.
**4.**	Sex	Was the researcher male or female?	Female.
**5.**	Experience and training	What experience or training did the researcher have?	FEK completed a 5 ECTS module entitled *Qualitative Inquiry* in University College Cork, Ireland and a course entitled *Introduction to Qualitative Research Methods* delivered by the University of Oxford. This course provides researchers with knowledge and skills in the application of qualitative research methods. This research study was the first qualitative research undertaken by FEK.
**Relationship with participants**			
**6.**	Relationship established	Was a relationship established prior to study commencement?	Some HCPs interviewed were known in a professional capacity to research team members FEK, LS,MB, MOD and SB.

**7.**	Participant knowledge of the interviewer	What did the participants know about the researcher (e.g. personal goals, reasons for doing the research)?	FEK had disclosed to all participants that she was a qualified pharmacist in Egypt and undertaking this study as part of her PhD, prior to conducting the interviews.
**8.**	Interviewer characteristics	What characteristics were reported about the interviewer/facilitator? (e.g. bias, assumptions, reasons and interests in the research topic)	FEK is an Egyptian pharmacist who was conducting this study as part of her PhD exploring the barriers and facilitators to medication management to myocardial infarction patients. This information was disclosed to participants ahead of the interview.
**Domain 2: Study design**			
**Theoretical framework**			
**9.**	Methodological orientation and theory	What methodological orientation was stated to underpin the study (e.g. grounded theory, discourse analysis, ethnography, phenomenology, content analysis)?	This study employed a descriptive qualitative approach as described by Sandelowski as its methodological orientation [2,3].
**Participant selection**			
**10.**	Sampling	How were participants selected (e.g. purposive, convenience, consecutive, snowball)?	Participants were identified and recruited through a combination of purposive, convenience, and snowball sampling. Professional contacts of study team members (LS, MB,MOD and SB) were purposively sampled to fulfil a minimum number of HCPs across various different characteristics (Table 1). Other HCPs were convenience sampled, and the study consent form and information leaflet were circulated via an administrator of the email list. Once an HCP completed an interview, they were requested to ask colleagues if they would be interested in

			participating and to share the colleagues contact details to the research team (snowball sampling).
**11.**	Method of approach	How were participants approached (e.g. face-to-face, telephone, mail, email)?	Five participants were recruited via email sent by LS, MOD and SB, and three participants who were professional contacts of the research team were purposively emailed by FEK. Two participants were emailed after other interviewees provided their contact details post-interview, and two participants contacted the research team after seeing the study advertisement.
**12.**	Sample size	How many participants were in the study?	12
**13.**	Nonparticipation	How many people refused to participate or dropped out? Reasons?	Three people refused to participate.

Setting

**14.**	Setting of data collection	Where were the data collected (e.g. home, clinic, workplace)?	Eleven interviews were conducted via videoconference using Microsoft Teams® and one was carried out in person and recorded using Microsoft Teams®. Participants were mainly located in their practice setting or at their home at the time of the interview.
**15.**	Presence of nonparticipants	Was anyone else present besides the participants and researchers?	No.
**16.**	Description of sample	What are the important characteristics of the sample (e.g. demographic data, date)?	Participant characteristics are shown in Table 1 of the manuscript. All interviews were conducted between December 2024 and May 2025.

**Data collection**			
**17.**	Interview guide	Were questions, prompts, guides provided by the authors? Was it pilot tested?	A topic guide (with prompts where appropriate) was developed based on a qualitative systematic review [4], the TDF, and the research team's knowledge and experience. The TDF is a validated framework that synthesises 33 behaviour change theories and is comprised of 14 domains based on 84 theoretical constructs related to behaviour change [5]. The topic guide was then iteratively refined during the study, where appropriate, to ensure that themes were explored in subsequent interviews.
**18.**	Repeat interviews	Were repeat interviews carried out? If yes, how many?	No.
**19.**	Audio/visual recording	Did the research use audio or visual recording to collect the data?	All interviews were audio-recorded. The recordings were stored securely and were subsequently deleted once the interviews were transcribed.
**20.**	Field notes	Were field notes made during and/or after the interview or focus group?	Field notes were recorded during each interview and were used to refine topic guides and inform data analysis.
**21.**	Duration	What was the duration of the interviews or focus group?	The mean duration of the interview was 41 minutes (range 30-50 minutes)
**22.**	Data saturation	Was data saturation discussed?	Thematic saturation was used as an indicator; base size was the number of interviews required to complete the sampling strategy outlined in Table 1. [6-8]
**23.**	Transcripts returned	Were transcripts returned to participants for comment and/or correction?	None.

**Domain 3: Analysis and findings**			
**Data analysis**			

**24.**	Number of data coders	How many data coders coded the data?	Two (FEK and LS).
**25.**	Description of the coding tree	Did authors provide a description of the coding tree?	Initially, non-hierarchical conventional codes were categorised and subsequently developed into themes. Each of the themes and their antecedent codes were then categorised under one of the predominant TDF domains identified during the directed content analysis.
**26.**	Derivation of themes	Were themes identified in advance or derived from the data?	Conventional content analysis consisted of open coding to inductively create initial, non-hierarchical codes. These initial codes were subsequently categorised to generate themes.<br><br>Directed content analysis was then employed whereby the transcripts were deductively coded using the TDF to identify the domains present.
**27.**	Software	What software, if applicable, was used to manage the data?	QSR NVivo® version 12.
**28.**	Participant checking	Did participants provide feedback on the findings?	No.
**Reporting**			
**29.**	Quotations presented	Were participant quotations presented to illustrate the themes/findings? Was each quotation identified? e.g. participant number	Yes.
**30.**	Data and findings consistent	Was there consistency between the data presented and the findings?	Quotations which support the findings are presented in of the results section in the manuscript.
**31.**	Clarity of major themes	Were major themes clearly presented in the findings?	Major themes are clearly presented in the results section of the manuscript as the predominant TDF domains.

**32.**	Clarity of minor themes	Is there a description of diverse cases or discussion of minor themes?	Subthemes are presented in the results section of the manuscript as conventional themes under each of the seven predominant TDF domains in the results section.

References:

1. Tong, A.; Sainsbury, P.; Craig, J. Consolidated Criteria for Reporting Qualitative Research (COREQ): A 32-Item Checklist for Interviews and Focus Groups. *International Journal for Quality in Health Care* **2007**, *19*, 349–357, doi:10.1093/intqhc/mzm042.
2. Sandelowski, M. Whatever Happened to Qualitative Description? *Research in Nursing & Health* **2000**, *23*, 334–340, doi:https://doi.org/10.1002/1098-240X(200008)23:4<334::AID-NUR9>3.0.CO;2-G.
3. Sandelowski, M. What's in a Name? Qualitative Description Revisited. *Res Nurs Health* **2010**, *33*, 77–84, doi:10.1002/nur.20362.
4. Hurley, E.; Walsh, E.; Foley, T.; Byrne, S.; Gleeson, L.; Dalton, K. GPs' Views of Pharmacist Services in General Practice: A Qualitative Evidence Synthesis. *Fam Pract* **2021**, 1–12, doi:10.1093/fampra/cmab114.
5. Cane, J.; O'Connor, D.; Michie, S. Validation of the Theoretical Domains Framework for Use in Behaviour Change and Implementation Research. *Implementation Science* **2012**, *7*, 37, doi:10.1186/1748-5908-7-37.
6. 29. Hennink, M.; Kaiser, B.N. Sample Sizes for Saturation in Qualitative Research: A Systematic Review of Empirical Tests. Soc. Sci. Med. 2022 , 292, 114523. https://doi.org/10.1016/j.socscimed.2021.114523.
7. Guest, G.; Namey, E.; Chen, M. A Simple Method to Assess and Report Thematic Saturation in Qualitative Research. PLoS ONE 2020, 15, e0232076. https://doi.org/10.1371/journal.pone.0232076.
8. Guest, G.; Bunce, A.; Johnson, L. How Many Interviews Are Enough?: An Experiment with Data Saturation and Variability. Field Methods 2006, 18, 59–82. https://doi.org/10.1177/1525822X05279903.
